# SPLUNC1 Regulates Cell Progression and Apoptosis through the miR-141-PTEN/p27 Pathway, but Is Hindered by LMP1

**DOI:** 10.1371/journal.pone.0056929

**Published:** 2013-03-05

**Authors:** Pan Chen, Xiaofang Guo, Houde Zhou, Wenling Zhang, Zhaoyang Zeng, Qianjin Liao, Xiayu Li, Bo Xiang, Jianbo Yang, Jian Ma, Ming Zhou, Shuping Peng, Juanjuan Xiang, Xiaoling Li, Colvin Wanshura LE, Wei Xiong, James B. McCarthy, Guiyuan Li

**Affiliations:** 1 Hunan Provincial Tumor Hospital and the Affiliated Tumor Hospital of Xiangya Medical School, Central South University, Changsha, Hunan, P.R. China; 2 Key Laboratory of Carcinogenesis of Ministry of Health and Key Laboratory of Carcinogenesis and Cancer Invasion of Ministry of Education, Cancer Research Institute, Central South University, Changsha, Hunan, P.R. China; 3 Hunan Key Laboratory of Nonresolving Inflammation and Cancer and Disease Genome Research Center, The Third Xiangya Hospital, Central South University, Changsha, Hunan, P.R. China; 4 Department of Laboratory Medicine and Pathology and Masonic Cancer Center, University of Minnesota, Minneapolis, Minnesota, United States of America; University of Alabama at Birmingham, United States of America

## Abstract

Little is known about the role of the host defensive protein short palate, lung and nasal epithelium clone 1 (SPLUNC1) in the carcinogenesis of nasopharyngeal carcinoma (NPC). Here we report that SPLUNC1 plays a role at a very early stage of NPC carcinogenesis. SPLUNC1 regulates NPC cell proliferation, differentiation and apoptosis through miR-141, which in turn regulates PTEN and p27 expression. This signaling axis is negatively regulated by the EBV-coded gene LMP1. Therefore we propose that SPLUNC1 suppresses NPC tumor formation and its inhibition by LMP1 provides a route for NPC tumorigenesis.

## Introduction

Nasopharyngeal carcinoma (NPC) is a nonlymphomatous, squamous cell carcinoma that occurs in the epithelial lining of the nasopharynx [1], [2]. NPC represents a significant clinical challenge in southern China and Southeast Asia, with an incidence of 15–50 per 100,000 [3]. NPC development is a multistage process influenced by complex interactions between genetic and environmental factors [4]. Chronic infection with Epstein–Barr virus (EBV) or other pathogens has been considered one of the most important factors NPC development [5], [6]. The EBV-encoded latent membrane protein 1 (LMP1) is an oncogene that is critically involved in the EBV-driven immortalization of B cells in vitro, and is able to transform rodent fibroblast and human cell lines in culture [7]. More than 90% of the adult population of the world have been infected by EBV, and although they become lifelong carriers of the virus, only about 0.03% of them develop NPC [8], which indicates that other factors exist to protect the EBV-infected nasopharyngeal epithelium against NPC.

Innate immune responses in epithelial cells provide a vital source of host defense molecules to protect against nasopharyngeal epithelial infection. These host defense molecules are natural immunobarriers against environmental threats with various mechanisms to bind, transport, cleave, or degrade microbes and their endotoxic byproducts [9], which decrease the risk of epithelial damage and the development of cancer. Short palate, lung and nasal epithelium clone 1 (SPLUNC1) was originally found to be downregulated in NPC and exhibits host defense properties [10], [11]. SPLUNC1 is secreted by large airway epithelial cells and has been shown to possess antimicrobial and anti-inflammatory functions [12], [13]. SPLUNC1 is able to induce apoptosis of EBV-infected B lymphocytes, and polymorphisms in the SPLUNC1 locus are associated with susceptibility to NPC [14]. SPLUNC1 also serves to protect the sodium channel protein (ENaC) from proteolytic cleavage, thus regulating airway surface liquid volume [15]. Effective maintenance of mucosal liquid volume, mediated by appropriate expression of SPLUNC1, is thought to promote mucociliary clearance of microorganisms from the airway [16]. While the involvement of SPLUNC1 in host defensive protein is well-delineated, its involvement in the tumorigenesis of NPC remains unclear.

Previous differential analysis of microRNA (miRNA) expression profiles after re-expression of SPLUNC1 in NPC cells showed that SPLUNC1 could decrease miR-141 expression in the highly tumorigenic and metastatic 5–8F NPC cells. PTEN has been validated as a miR-141 target by 3′-untranslated region (3′-UTR) luciferase reporter assays [17]. While the extent of PTEN involvement in NPC tumorigenesis is an area of current investigation, loss of PTEN function through deletion, mutation, and/or decreased expression has been found in numerous human sporadic cancers [18], [19] and in hereditary cancer syndromes [20], [21]. Furthermore, activation of PI3K/AKT and other signalling pathways by LMP1 leads to enhance NPC cell growth and migration [22]–[25]. Together, these data indicate that crosstalk between the SPLUNC1-miR-141-PTEN and LMP1-Akt signaling axes may be important in NPC development and progression. In the present study, we have determined that the host defense protein SPLUNC1 regulates NPC cell apoptosis, proliferation, and differentiation through the miR-141-PTEN/p27-Akt pathway, and this activity of SPLUNC1 is negatively regulated by the EBV-coded gene LMP1.

## Results

### SPLUNC1 can Inhibit EBV Infection of Human Peripheral Lymphocytes

**Figure 1:**
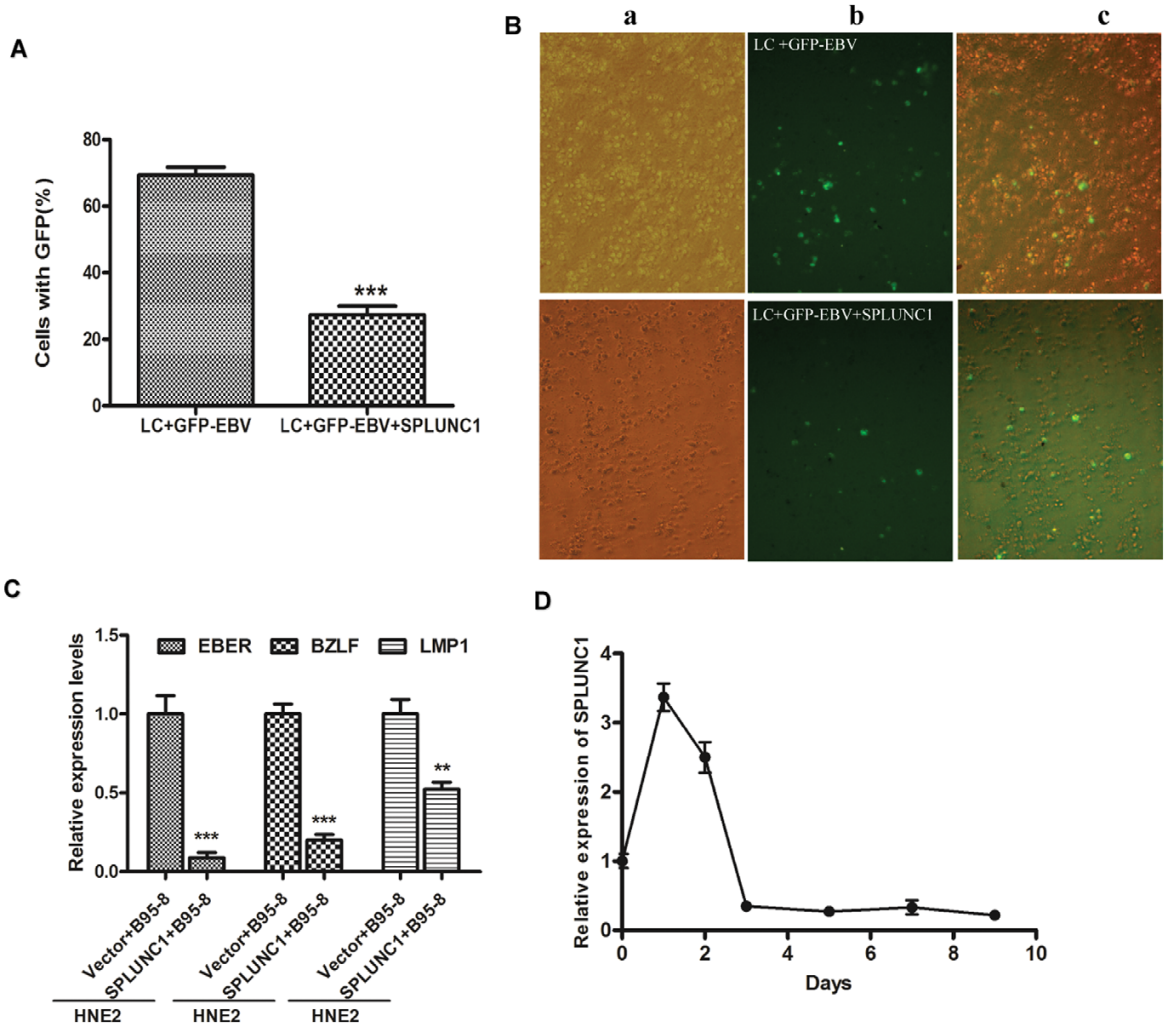
SPLUNC1 inhibited the course of EBV infection. (**A, B**) Human peripheral blood lymphocytes (LCs) were infected with green fluorescence protein (GFP)-tagged EBV. Approximately 70% of LCs showed green fluorescence after infection, but only 28% of LCs treated with the recombinant SPLUNC1 protein showed green fluorescence. (**C, D**)HNE-2 cells were co-cultured with B95-8 cells, which product EBV, for 1, 2, 3, 5, and 7 days, and B95-8 cells were then removed by complement-activated cellular cytotoxicity test. (**C**) Expression of EBER, BZLF1 and LMP1 was lower in the presence of enforced SPLUNC1 expression versus the control in HNE-2 cells. (**D**) SPLUNC1 expression in the HNE-2 cells was increased significantly 1 day after the addition of B95-8 cells to the HNE-2 culture system, and then decreased. After 3 days of co-culture with B95-8, SPLUNC1 expression decreased to its lowest level.

To determine how expression of SPLUNC1 influences EBV infectivity, the rate of infection and expression of EBV-encoded genes were analyzed. To evaluate the direct effect of SPLUNC1 on EBV infectivity, a green fluorescence protein (GFP)-tagged EBV was designed to facilitate monitoring the course of infection. The proportion of human peripheral lymphocytes (LC) infected by GFP-EBV was reduced when cells were treated with recombinant SPLUNC1 protein than the control, indicating that expression of SPLUNC1 can prevent EBV entry into human lymphocytes (**Figs. 1A and 1B**). HNE-2 cells were then co-cultured with EBV for 1, 2, 3, 5, and 7 days, and B95-8 cells were removed by complement-activated cellular cytotoxicity test. Expression of the EBV-encoded genes EBER, BZLF1, and LMP1 was much lower in SPLUNC1-expressing cells than in vector control HNE-2 cells (**Figs. 1C and 1D**). SPLUNC1 expression in the HNE-2 cells was increased significantly 1 day after addition of the B95-8 cells to the HNE-2 culture system, and then decreased; after co-culture with B95-8 for 3 days, SPLUNC1 expression reached its lowest level (**Fig. 1D**).

### SPLUNC1 Expression is lost during NPC Progression

**Figure 2:**
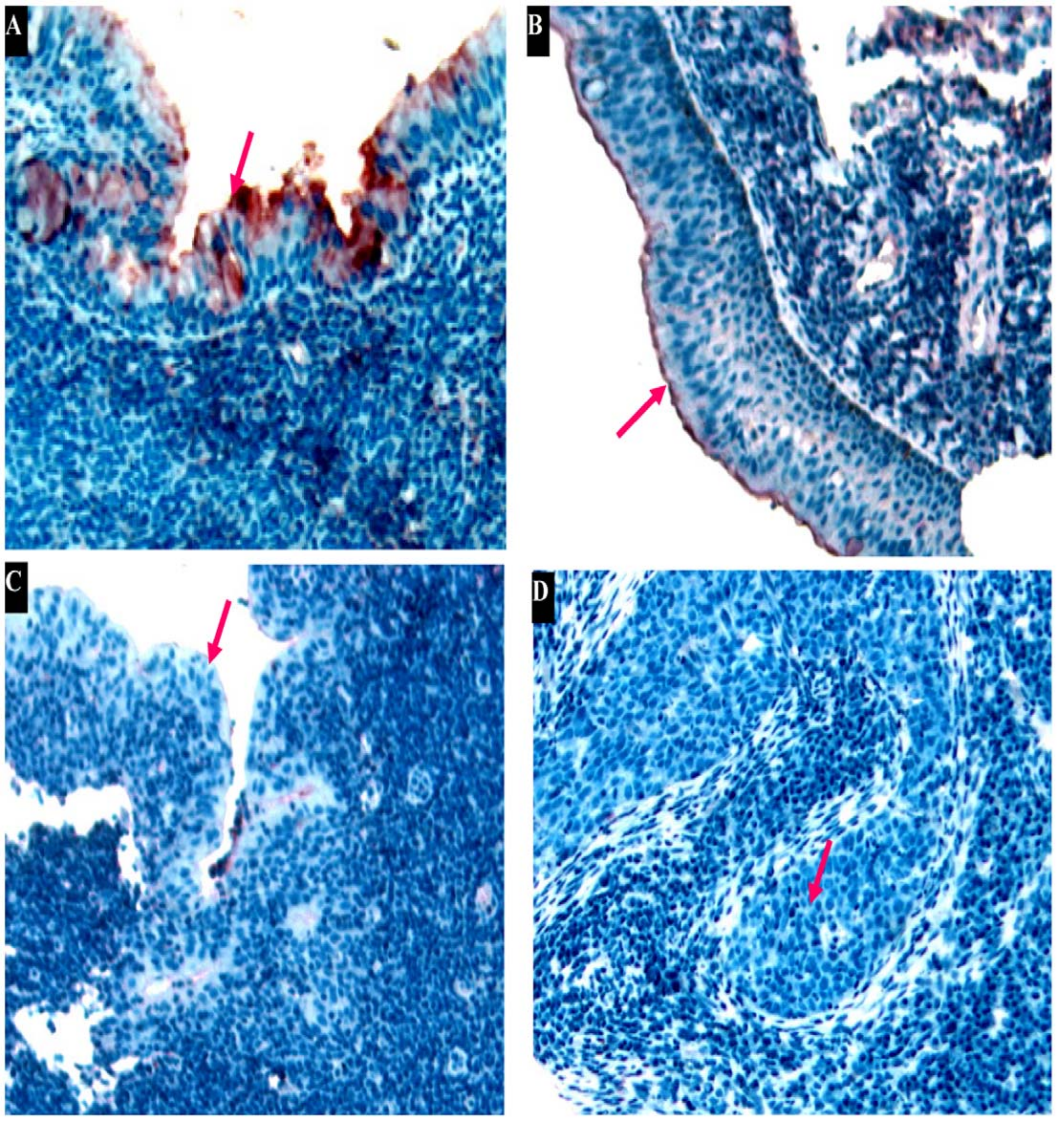
SPLUNC1 protein expression was decreased in highly differentiated nasopharyngeal carcinoma. (**A**) SPLUNC1 protein was high expressed in normal human nasopharyngeal epithelium, SPLUNC1 expression was gradually downregulated with progression from (**B**) mild to (**C**) severe atypical hyperplasia of nasopharyngeal tissues. (**D**) No positive staining for SPLUNC1 was observed in the epithelium of nasopharyngeal carcinoma.

As shown in Table 1, immunohistochemical analysis revealed the absence of SPLUNC1 expression in 93.85% of newly diagnosed NPC tissue specimens, weakly positive expression of SPLUNC1 in 6.15%, and no evidence of positive or strongly positive expression. In glands adjacent to the NPC, positive or strongly positive SPLUNC1 expression was not observed, but weakly positive SPLUNC1 expression was observed in 54.05% of glands. All normal nasopharyngeal tissue specimens were positive for SPLUNC1 expression, 76.47% of them were highly expressed SPLUNC1. Following radiotherapy for NPC, SPLUNC1 expression was significantly upregulated. With progression from mild to severe atypical hyperplasia of nasopharyngeal tissue, SPLUNC1 expression was downregulated (**Fig. 2**). Nevertheless, 3.39% of atypical hyperplasia tissue specimens were positive or strongly positive for SPLUNC1.

**Table 1:** SPLUNC1 expression in nasopharyngeal biopsy specimens at various stages.

Classification	SPLUNC1 expression level		
	Negative	Weakly expression	High expression
Normal nasopharynx	0	23.53% (4 of 17)	76.47% (13 of 17)
Chronic inflammation of nasopharyngeal mucosa	0	4.62% (3 of 65)	95.38% (62 of 65)
Atypical hyperplasia	47.46% (28 of 59)	49.15% (29 of 59)	3.39% (2 of 59)
Newly diagnosed NPC	93.85% (183 of 195)	6.15% (12 of 195)	0
Epithelium adjacent to NPC	34.74% (33 of 95)	53.68% (51 of 95)	11.58% (11 of 95)
Glandular adjacent to NPC	45.95% (17 of 37)	54.05% (20 of 37)	0
NPC after radiotherapy	34.78% (8 of 23)	34.78% (8 of 23)	30.44% (7 of 23)

### Pairwise Association between Abnormal Expression of EBER-1 Hybridization Signals, LMP1 and SPLUNC1 in NPC

The expression of the EBER-1 hybridization signals and LMP1 in NPC was 71.2% and 78.0% respectively. In NPC, the expression of LMP1 was strongly positive correlated with the EBER-1 hybridization signals (r = 0.204, *P* = 0.001) and negative correlated with SPLUNC1 protein expression (r = −0.136, *P* = 0.028).

### miR-141 is Regulated by SPLUNC1 and Related to the Differentiation Stages of NPC Cell Lines

**Figure 3:**
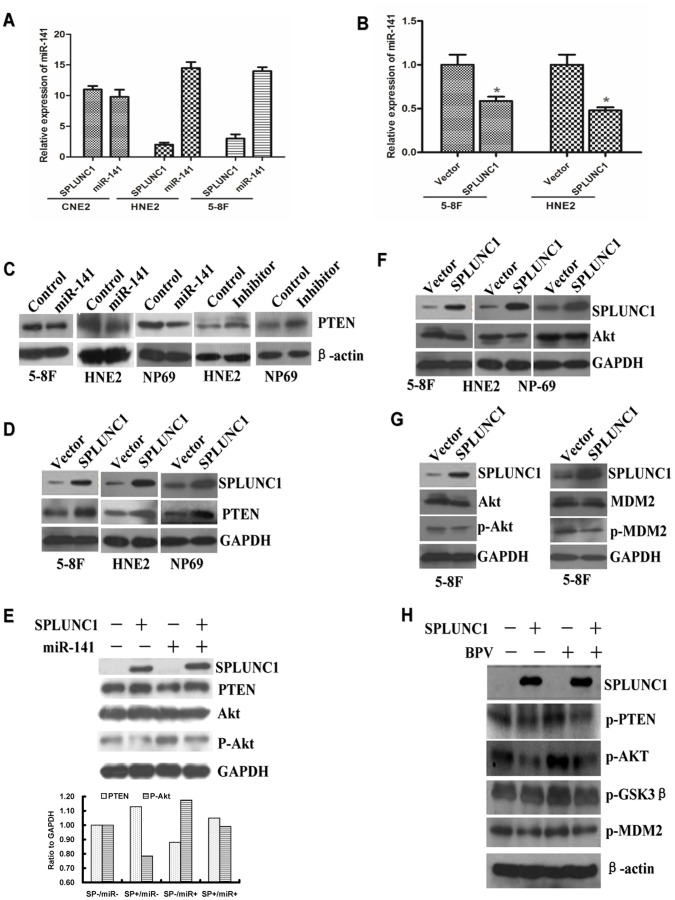
SPLUNC1 inhibited miR-141 expression and the effects of miR-141 and SPLUNC1 on PTEN expression in nasopharyngeal carcinoma cells. (**A**) SPLUNC1 expression in CNE-1 nasopharyngeal carcinoma cells was higher than that in the HNE-2 and 5–8 F cells, while the miR-141 expression in CNE-1 cells was lower than that in the HNE-2 and 5–8 F cells (P<0.05). (**B**) When SPLUNC1 was overexpressed in HNE-2 and 5–8 F cells, miR-141 expression was decreased (P<0.05). (**C**) Expression of miR-141 mimics in NPC cells decreased the expression of PTEN; expression of miR-141 inhibitor increased the expression of PTEN. (**D**) Enforced expression of SPLUNC1 in NPC cells increased PTEN expression. (**E**) miR-141 mimics inhibited PTEN expression and increased Akt phosphorylation in the presence of enforced SPLUNC1-expression in NPC 5–8 F cells. (**F**) Enforced expression of SPLUNC1 decreased Akt expression in NPC cells; and decreased the levels of phosphorylated Akt and MDM2 (**G**), but had little effect on GSK3β. SPLUNC1 inhibited the baculovirus phosphatase (BVP)-induced phosphorylation of PTEN and Akt(**H**).

Because differentiation stage can influence miRNA expression, miR-141 expression was detected in highly, poorly and undifferentiated NPC cell lines. SPLUNC1 expression was increased in the highly differentiated cell line CNE-1 as compared to the poorly differentiated HNE-2 and undifferentiated 5–8 F cell lines. The cell line with high SPLUNC1 expression, CNE-1, also displayed low miR-141 expression (**Fig. 3A**). In the poorly differentiated and undifferentiated cell lines, SPLUNC1 expression was decreased and miR-141 expression was increased. Induced expression of SPLUNC1 in HNE-2 and 5–8 F cells led to decrease expression of miR-141 (**Fig. 3B**).

### SPLUNC1 Regulates the Akt Pathway through PTEN and Inhibits the Baculovirus Phosphatase-induced PTEN Phosphorylation in NPC Cells

PTEN was previously identified as one of the target genes of miR-141 [17]. When synthesized miR-141 mimics was transfected into the 5–8 F, HNE-2, and an immortalized normal nasopharyngeal epithelial cell line NP-69, the expression of PTEN protein was significantly decreased; if these cells were transfected with a miR-141 inhibitor, PTEN expression was increased (**Fig. 3C**). Enforced expression of SPLUNC1 in these cells also upregulated PTEN expression **(** **Fig. 3D**), and co-expression of miR-141 with SPLUNC1 was able to reverse the increase in PTEN protein expression induced by SPLUNC1 alone (**Fig. 3E**). Furthermore, Akt expression and phosphorylation downstream of PTEN were inhibited by enforced expression of SPLUNC1 in the highly metastatic 5–8 F cells (**Fig. 3F**). Phosphorylation of the AKT target MDM2 was also inhibited by SPLUNC1 (**Fig. 3G**).

PTEN inhibits its downstream signal cascade by self dephosphorylation, and this dephosphorylation can be inhibited by a baculovirus phosphatase (BVP), which induces the phosphorylation of PTEN and activates the Akt pathway [26]. HNE-2 cells treated with BVP exhibited increased phosphorylation of PTEN, Akt and MDM2; however enforced expression of SPLUNC1 was able to inhibit this activity of BVP (**Fig. 3H**).

### SPLUNC1 Promotes NPC Cell Apoptosis

**Figure 4:**
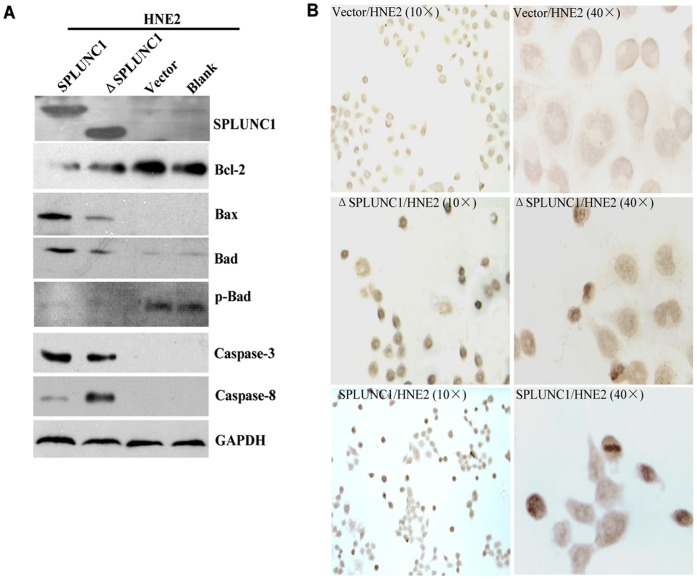
SPLUNC1 induced cellular apoptosis. (**A**) SPLUNC1 and the BPI domain-deleted mutant, ΔSPLUNC1, increased the expression of the proapoptotic proteins BAX, BAD caspase-3 and 8, and decreased the antiapoptotic protein Bcl-2 expression. (**B**) Enforced expression of SPLUNC1 and ΔSPLUNC1 in HNE-2 cells increased cellular apoptosis.

As most NPCs are poorly differentiated [27], the poorly differentiated cell line HNE-2 was selected for further studies. As Akt pathway signaling can lead to apoptosis, we next sought to determine the effect of SPLUNC1 on the expression and activation of pro-apoptotic proteins. Enforced expression of SPLUNC1 and its BPI domain deletion mutant (ΔSPLUNC1) decreased expression of the anti-apoptotic protein Bcl-2 (**Fig. 4A**). Enforced expression of SPLUNC1 also induced expression of the proapoptotic proteins BAX, BAD, and caspases-3 and 8, and decreased the phosphorylation of BAD at serine 136 **(** **Fig. 4A** **)**. ΔSPLUNC1 was able to increase the expression of the proapoptotic proteins **(** **Fig. 4A** **)**, indicating that the BPI domain is not required for SPLUNC1-mediated induction of pro-apoptotic signaling events. Apoptosis was further validated by terminal deoxynucleotidyl transferase biotin-dUTP nick end labeling (TUNEL) analysis in HNE-2 cells; approximately 28% of cells stably transfected with SPLUNC1 and 22% of the ΔSPLUNC1-expressing cells stained positive, most of which showed pyknosis or karyorrhexis and apoptotic bodies **(** **Fig. 4B** **)** versus 3.0% of untransfected and 3.5% of control vector-transfected cells **(** **Fig. 4B** **)**. Together, these results indicate that SPLUNC1 can induce apoptosis in HNE-2 NPC cells.

### SPLUNC1 Regulates Cell Differentiation and Growth Inhibition via MAPK and the p27 Pathway

**Figure 5:**
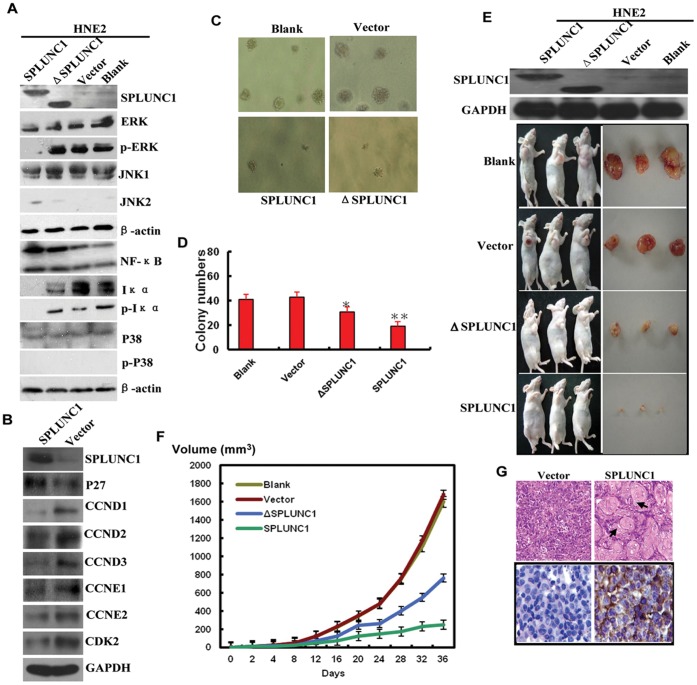
SPLUNC1 promoted cell differentiation and inhibits tumor growth. (**A**) Enforced expression of SPLUNC1 increased JNK2 and NFκB protein expression, and inhibited phosphorylation of ERK (p-Tyr-204) and Iκα (Ser-32). ΔSPLUNC1 had no effect on phosphorylation of ERK (p-Tyr-204) and Iκα (Ser-32). (**B**) SPLUNC1 also increased p27 expression and decreased the expression of CCND1, CCND2, CCND3, CCNE2, and CDK2. (**C, D**) clone formation in soft agar; tumors formed in the presence of full length SPLUNC1 or ΔSPLUNC1 were smaller versus control. (**E, F**) SPLUNC1 and ΔSPLUNC1 inhibited tumor formation in nude mice and (**G**) SPLUNC1 expression in the transplant tumor was validated by immunohistochemical analysis.

As PTEN, along with MAPK and p27, are very important regulators of cell differentiation, the effects of SPLUNC1 on the MAPK and p27 pathways were assessed. While enforced expression of SPLUNC1 had no effect on the expression of JNK1, p38 and ERK, it did increase JNK2 expression and inhibited ERK (Tyr-204) phosphorylation in HNE-2 cells **(** **Fig. 5A** **)**. ΔSPLUNC1 was unable to induce JNK2 expression or inhibit ERK (Tyr-204) phosphorylation **(** **Fig. 5A** **)** which implicates the BPI domain doesn’t involve in MAPK pathway regulation.

Because the expression of miR-141 has been shown to result in G_1_ arrest supported by increased p27/kip1 expression in breast cancer cells [28], we next sought to determine whether this signaling axis is intact in NPC cells. In support of a role for SPLUNC1 in cell cycle regulation, enforced expression of SPLUNC1 significantly induced p27 protein expression **(** **Fig. 5B** **)**. The cell cycle proteins cyclin D1 (CCND1), CCND2, CCND3, CCNE2, and cyclin-dependent kinase 2 (CDK2) were also decreased in the presence of SPLUNC1 **(** **Fig. 5B** **)**.

To determine the ability of SPLUNC1 to induce tumor cell transformation in vitro, HNE-2 cells stably expressing SPLUNC1 or control vector were grown in soft agar. SPLUNC1-expressing cells formed fewer and smaller colonies than control cells (**Fig. 5C and 5D**). To directly evaluate the role of SPLUNC1 in tumor formation in vivo, groups of nude mice were injected subcutaneously with cells stably transfected with SPLUNC1, ΔSPLUNC1 or vector control. After 4 weeks, only 6 of the 10 mice injected with SPLUNC1-expressing cells had developed very small tumors versus the vector control (**Fig. 5E and 5F**). ΔSPLUNC1 was also able to inhibit tumor growth; however tumors derived from ΔSPLUNC1-expressing cells were bigger than those from SPLUNC1-expressing cells (**Fig. 5E and 5F**). Interestingly, tumors derived from SPLUNC1-expressing cells revealed many intercellular bridges and keratin pearls, which are markers of highly differentiated NPC lesions (**Fig. 5G**). In contrast, tumors produced by control vector-transfected cells revealed low to moderate differentiation (**Fig. 5G**). Together, these data implicate SPLUNC1 involved in both in vitro and in vivo NPC tumorigenesis and tumor differentiation.

### SPLUNC1 Regulation of the PTEN/Akt Pathway can be Hindered by LMP1

**Figure 6:**
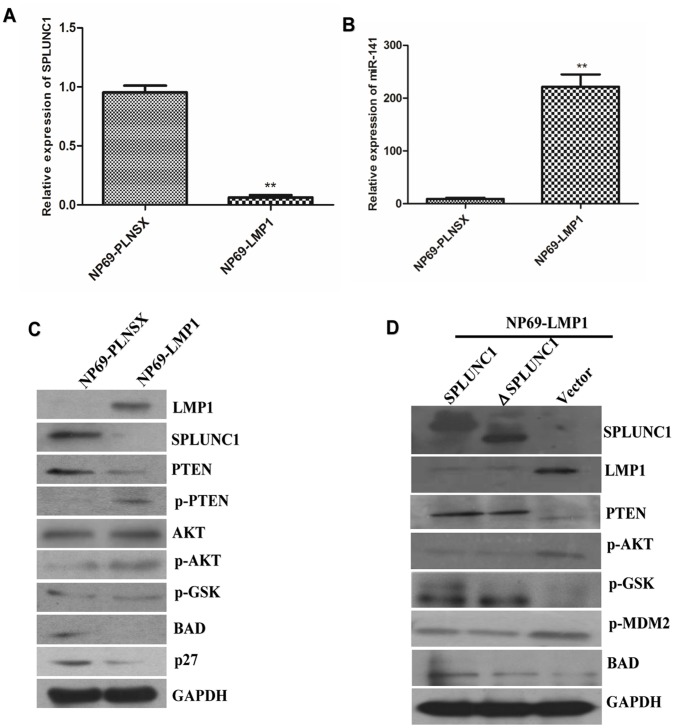
The interaction between SPLUNC1 and LMP1. (**A**) SPLUNC1 expression was significantly decreased in NP-69 cells transfected with LMP1. (**B**) miR-141 expression was significantly increased in NP-69 cells transfected with LMP1. (**C**) PTEN expression was decreased and the Akt pathway was activated by foreign LMP1 expression. (**D**) Re-expression of SPLUNC1 in LMP1-transfected cells led to inhibition of LMP1 expression and induction of PTEN.

As an innate immune defense protein on the surface of epithelium, SPLUNC1 must fight with the virus and its product. Since EBV and LMP1 play critical roles in NPC, the effect of SPLUNC1 on EBV and LMP1 was investigated using the EBV and LMP1-free human nasopharyngeal epithelium cell line NP-69. SPLUNC1 mRNA expression was significantly decreased in NP-69 cells transfected with LMP1 (**Fig. 6A**), while miR-141 expression was increased significantly in the cells transfected with LMP1 (**Fig. 6B**). PTEN protein expression was decreased and Akt phosphorylation was increased in the presence of enforced LMP1 expression (**Fig. 6C**). But re-expression of SPLUNC1 could reverse this inhibition of PTEN expression and Akt phosphorylation by LMP1 in the NP69 cell line, which was independent of the BPI domain of SPLUNC1 **(** **Fig. 6D** **)**. Together these data indicate that EBV-encoded LMP1 can suppress SPLUNC1-mediated signaling events through dual control of SPLUNC1 and miR-141 expression.

## Discussion

Environmental risk factors and genetic susceptibility play important roles in the pathogenesis of NPC [29]–[31]. In the past few years, studies on the genetic susceptibility have identified several susceptibility loci [32], [33], and many NPC candidate susceptibility genes have been described [34]–[36]. However, few of the genes identified have been related to EBV infection and EBV-mediated tumorigenesis, although EBV infection has been widely considered the most important environmental risk factor for the NPC. While previous studies have shown SPLUNC1 play a role in host defense [10], [14], the depth of involvement of SPLUNC1 in NPC tumorigenesis remains to be determined.

Here we have shown that the host defense SPLUNC1 functions as a putative tumor suppressor gene in NPC first by preventing EBV infection. Furthermore, its expression is lost during human NPC development: while it is expressed in untransformed and hyperplasic nasopharyngeal tissue, its expression is lost in the majority of NPC patient samples. At a cellular level, SPLUNC1 then promotes NPC cell apoptosis and differentiation while limiting cell cycle progression and growth via negative regulation of miR-141 expression, relieving suppression of PTEN and p27 expression. Altogether our data implicate SPLUNC1 as an important tumor suppressor protein in EBV-driven NPC.

EBV is widely considered the most important biological risk factor for NPC through its integration of viral oncogenes into the host cells [37]. We demonstrated that SPLUNC1 inhibited the entry of GFP-EBV into the lymphocytes. Once the EBV made contact with the epithelial cells, the nasopharyngeal epithelial cells induced SPLUNC1 expression. SPLUNC1 expression, in turn, suppressed expression of the EBV-encoded genes EBER, BZLF and LMP1. Based on these data, we propose a model in which the absence of SPLUNC1 creates a permissive environment for EBV infection and expression of the viral oncogene LMP1. At this stage, LMP1 inhibits the expression of SPLUNC1, and the tumor suppressive role of SPLUNC1 through the miR-141/PTEN/p27 pathway is compromised, promoting NPC progression. Interestingly, we found that if SPLUNC1 was re-expressed at this time, the oncogenicity of LMP1 was impaired. Therefore, SPLUNC1 plays important roles both in the prevention of EBV infection and in EBV-mediated NPC development; it may therefore be a candidate target gene for the development of anticancer therapies in NPC.

Based on our findings, we propose that SPLUNC1 plays a role at an early stage of tumorigenesis. We found SPLUNC1 to be expressed in most of the normal nasopharyngeal epithelial specimens, but it was decreased slightly in nasopharyngeal epithelium with mild atypical hyperplasia. With progression from mild to severe atypical hyperplasia of nasopharyngeal tissue, SPLUNC1 expression was reduced significantly, and absence of SPLUNC1 expression was found in 93.85% of newly diagnosed NPC tissue specimens. These results further verified that SPLUNC1 plays a role at the very earliest stage of NPC carcinogenesis, even before the development of mild atypical hyperplasia. Because SPLUNC1 is a secretory protein, it is easily detected in nasopharyngeal lavage fluid; we therefore propose that SPLUNC1 may be an excellent prognostic and early diagnostic marker for NPC.

While our results indicate that SPLUNC1-mediated suppression of miR-141 plays a role in PTEN-mediated AKT activation and that loss of SPLUNC1 occurs in later stages of NPC, Chen et al. found that miR-141 was not differentially expressed in a miRNA profile of NPC samples as compared to adjacent normal tissues [38]. As miRNA plays different roles dependent on stage, cancer type, and other clinical variables, we hypothesized that miR-141 expression in adjacent tissues had increased to a level similar to that in the NPC tissues and that the increase in miR-141 expression might be an early event in the development of NPC or related to cellular differentiation in NPC. In support of this hypothesis, we found SPLUNC1 expression to be decreased in a highly differentiated cell line as compared to an undifferentiated cell line, while miR-141 expression increased. Therefore, we hypothesized that SPLUNC1 and miR-141 were highly related to cellular differentiation in NPC.

The present study elucidates a mechanism by which SPLUNC1-induced miR-141 contributes to NPC progression. To discover the mechanism by which SPLUNC1 and its target miR-141 regulated cell differentiation, it was first validated that PTEN was downregulated by miR-141, and that SPLUNC1 could increase PTEN expression. We further demonstrated that the downregulation of PTEN expression by SPLUNC1 was partially dependent on miR-141.

Furthermore, we demonstrated that expression of SPLUNC1 and miR-141 decreased PTEN and Akt phosphorylation. Re-expression of SPLUNC1 in HNE-2 cells was sufficient to inhibit BVP-induced AKT expression and phospnorylation. This suggests that SPLUNC1 can efficiently enhance the dephosphorylation of PTEN and inhibit the PI3K/Akt signal pathway. We further found that SPLUNC1 was able to enhance apoptosis of HNE-2 cells and activate BAX, caspase-3 and caspase-8 even when the BPI domain was deleted, suggesting that the BPI domain is not involved in AKT pathway-mediated apoptosis.

Another important target of miR-141 is the cyclin-dependent kinase inhibitor 1B/p27. Many studies have shown p27 accumulate in the presence of PTEN and to act cooperatively during tumorigenesis [39]. In accord with these studies, we have found p27 expression to be partially inhibited following inactivation of the PTEN pathway by BVP. Although BVP was able to promote cell growth and proliferation of NPC cells, enforced expression of SPLUNC1 in these cells negated these effects of the BVP, indicating that SPLUNC1-mediated inhibition of miR-141 expression is capable of rescuing PTEN pathway inhibition.

The MAPK pathway is an important pathway in the regulation of cell differentiation [40], although SPLUNC1 and truncated ΔSPLUNC1 had no effect on the total JNK1, p38, and ERK expression, it did clearly inhibit ERK phosphorylation and enhance JNK2 expression, leading to differentiation of NPC cells. This was validated by histological analysis of the transplant tumors in nude mice: the transplant tumors of the SPLUNC1/HNE-2 cells revealed high levels of differentiation; while the transplant tumors of the vector/HNE-2 cells remained at a low differentiation status.

This research has demonstrated that the host defensive protein SPLUNC1 acts as a tumor suppressor gene in the formation of EBV-driven NPC. As a tumor suppressor, its action has two fold: first, it prevents EBV entry and protein expression; second, it inhibits the growth of NPC cells, and enhances cellular apoptosis and differentiation. These cellular effects of SPLUNC1 are mediated principally by miR-141, which in turn regulates PTEN and p27 expression. In addition to direct regulation by miR-141, p27 can also be regulated by PTEN. These findings critical insight into the mechanism of EBV-mediated NPC development and implicate that SPLUNC1 could be exploited in the development of targeted therapies and serve as a diagnostic and prognostic factor for NPC.

## Materials and Methods

### Ethics Statement

Before study initiation, ethical approval was obtained from the Cancer Hospital of the Hunan province in Changsha, and the Central South University Ethics Review committees/Institutional Review Boards. NPC samples, nontumor nasopharyngeal epithelial tissues as well as peripheral blood lymphocytes from normal volunteers were collected at the Cancer Hospital of the Hunan province. Written informed consent was obtained from all patients and volunteers.

All animal procedures were conducted in accordance with protocols approved by the Institutional Animal Care and Use Committee (IACUC) of Central South University. Animals were allowed access to standard chow diet and water ad libitum and were housed in a pathogen-free barrier facility with a 12L:12D cycle. The mice were sacrificed by CO_2_ asphyxiation.

### Plasmid and miRNA

The coding region of the SPLUNC1 gene and its BPI domain (aa 110–238)-deleted mutant (ΔSPLUNC1) were inserted into the pEGFP vector as previously described [41]. The plasmid p2089 (Maxi-EBV), which contained the complete EBV genome of the B95-8 strain was kindly provided by Professor Hammerschmidt (GSF-National Research Center for Environment and Health, Germany). Two eukaryotic expression vectors with the EBV-related BZLF1 and BALF4 genes (pcDNA3.1 (+)/BZLF1 and pcDNA3.1 (+)/BALF4) were constructed as previously described [42]. miR-141 mimics (141 M), negative control (NC) mimics, miR-141 inhibitors (141 I), and negative control (INC) inhibitors were purchased from Shanghai Gene-Pharma Co (Shanghai, China).

### Tissue Array and Immunohistochemistry

Tissue array was constructed as described in our previous study [43]. The tissue core numbers on each section were slightly different because of additional losses suffered from block trimming and staining procedures. 17 normal nasopharyngx, 65 chronic inflammation of nasopharyngeal mucosa, 59 atypical hyperplasia, 195 newly diagnosed NPC, 95 epithelium adjacent to glandular adjacent to NPC, and 37 NPC after radiotherapy were placed in one TMA block. Immunohistochemistry (IHC) was performed using the peroxidase antiperoxidase technique after a microwave antigen retrieval procedure [6], [44], [45]. The sections were incubated at 4°C overnight using mouse anti-human SPLUNC1 antibody (1∶200, Abcam, MA). A semiquantitative scoring criterion for IHC was used, in which both staining intensity and positive areas were recorded.

### Cell Culture and Transfection

Three human NPC lines, CNE-1 with high differentiation, HNE-2 with low-differentiation, and the undifferentiated 5–8 F, were cultured in RPMI-1640 media (Invitrogen, Carlsbad, CA, USA) supplemented with 10% fetal bovine serum (FBS; Invitrogen). An immortalized normal nasopharyngeal epithelial cell line NP-69, NP-69 cells stably transfected with LMP1 (NP69-LMP1), and NP-69 cells transfected with vector alone (NP69-PLNSX) were maintained in keratinocyte serum-free medium (Invitrogen). PEGFP-SPLUNC1, pEGFP-ΔSPLUNC1, pEGFP control plasmids, and short interfering RNA (siRNA) duplex homologs in sequence with 141 M, 141 I, NC, and INC were transfected into CNE-1, HNE-2, 5–8 F and NP-69 cells using Lipofectamine 2000 (Invitrogen) according to the protocol of the manufacturer. To detect the effects of SPLUNC1 on LMP1-overexpressed cells, NP69-LMP1 cells were also transfected with pEGFP-SPLUNC1, pEGFP-ΔSPLUNC1, and pEGFP control plasmids. HEK-293 cells stably transfected with p2089 plasmid (293-EBV) were maintained in DMEM medium with 10% FBS.

### RNA Isolation and Real-time PCR Analysis

Total RNA was isolated using TRIzol reagent (Invitrogen). A reverse transcription reaction was performed using a Fermentas Revert Aid First Strand cDNA Synthesis kit and real-time quantitative PCR (qPCR) was performed using an IQ5 Multicolor Detection System (Bio-Rad). Glyceraldehyde-3-phosphate dehydrogenase (GAPDH) was used as an internal control. The relative changes in expression were calculated using the 2^−ΔΔ^CT (where CT is threshold cycle) method. The primers for real-time PCR analysis were followings: GAPDH forward 5′-ATCAAGATCATTGCTCCTCCTGAG-3′, GAPDH reverse 5′-CTGCTTGCTGATCCACATCTG-3′, SPLUNC1 forward 5′-CCCA TTCAAGGTCTTCTGGA-3′, SPLUNC1 reverse 5′-CTGTAGTCCGTGGATCAGCA-3′, EBER forward 5′-AGGACCTACGCTGCCCTA-3′, EBER reverse 5′-AAAACATGCG GACCACCA-3′, BZLF1 forward 5′-CATGTTTCAACCGCTCCGACTGG-3′, BZLF1 reverse 5′-GCGCAGCCTGTCATTTTCAGATG-3′, CAJA-DRBI forward 5′-CGTTTCT TGGAGTATAGC-3′, CAJA-DRBI reverse 5′-CACTAGGAACCTCTCTGA-3′.

### Antibodies and Western Blot Analysis

Antibodies specific for human PTEN, Akt, GSK3β, MDM2, Bcl-2, BAD, caspase-3, caspase-8, ERK, JNK, NFκB, Iκα, p38, p27, CCND1, CCND2, CCND3, CCNE1, CCNE2, CDK2, β-actin, GAPDH, and Phospho-PTEN, Phospho-Akt, Phospho-GSK3β, Phospho-MDM2, Phospho-BAD, Phospho-ERK, Phospho-p38, and Phospho-Iκα were purchased from Cell Signal Inc (Danvers, MA) and R&D system. Antibody for SPLUNC1 was previously produced by us. Anti-LMP1 mouse monoclonal antibody was purchased from Dako (Carpinteria, CA). HRP-labeled goat antirabbit, mouse and rabbit antigoat secondary antibodies were purchased from Sigma (Shanghai, China).

### Tumor Formation Assay in Nude Mice

Male BALB/c nude mice at 4–6 weeks of age were used and divided into two groups 10 mice (per group). To assess the effect of SPLUNC1 on tumorigenicity in vivo, 5×10^6^ pEGFP-C2-SPLUNC1 or pEGFP-C2 plasmids transfected HNE2 cells were subcutaneously injected in the flank of the mice, respectively. Three dimensions of the tumors were measured every four days. The mice were sacrificed after 6 weeks, and photographed and weight in each groups.

### Generation of Green Fluorescence Protein-labeled EBV

The viral genes BZLF1 and BALF4 in the pcDNA3.1 (+) vector were transiently transfected into the 293-EBV cells, and the EGFP-EBV was released from the transfected cells into the supernatant because the expression of BZLF1 and BALF4 induced lytic replication of the 293-EBV cells. The supernatant was then collected and filtered through a 0.45-mm pore-size filter, the green fluorescence protein-labeled EBV (GFP-EBV) was stored at −70°C.

### Isolation of Human Peripheral Blood Lymphocytes and Infection with Green Fluorescence Protein-labeled EBV

Peripheral blood lymphocytes (LCs) were isolated from venous blood of three volunteers using density gradient centrifugation in Lympholyte-H (Cedarlane, Ontario, Canada) according to the instructions of the manufacturer. LCs obtained from the three volunteers were divided into three groups: one group was cultured in RPMI-1640 with 10% heat-inactivated FBS; the second group was treated with GFP-EBV; the third group was treated with GFP-EBV and 5 µg/ml human recombinant SPLUNC1, incubated at 37°C for 48 hours, and then re-fed with fresh medium. On day 3 postinfection, the infection efficiency was determined by counting the ratio of cells with green fluorescence using the fluorescence microscope.

### Co-culture of EBV and Nasopharyngeal Carcinoma HNE-2 Cells

HNE-2 cells were seeded into 6-well culture plates (2×10^5^ cells/well). For preparation of the viral donors, B95-8 cells were cultured in RPMI-1640 medium until the exponential growth phase (cell density: 10^7^ cells/ml) was reached. At that point, 2 ml B95-8 suspension (with EBV) was applied (8 ml/well) to the HNE-2 epithelial cells at 37°C for 1, 2, 3, 5, and 7 days, and cells were then washed thoroughly four or five times with PBS. A B95-8 cell-specific gene fragment of the callithrix jacchus MHC class II antigen (CAJA-DRB1) was amplified by PCR in HNE-2 cells to validate that the B95-8 cells were removed completely from the co-culture system. RNA was isolated from the treated HNE-2 cells and SPLUNC1 expression was analyzed by real-time RT-PCR. Data are represented as mean ± SEM.

### Statistical Analysis

Significant difference between gene expression by real-time RT-PCR analysis, cell survival, and proliferation were analyzed by ANOVA analysis and the results were expressed as a mean ± standard deviation (SD). Immunohistochemistry and in situ hybridization staining of the TMA sections were scored microscopically at a magnification of ×400 in all the available tumors and epithelial tissues that met the typical morphological criteria of the two pathologists. Scores were then analyzed by the χ^2^ test or Fisher’s exact test using EPI software (EPI Info, version 3.2.2, www.CDC.gov/epiinfo/). A P value <0.05 was considered statistically significant, and all statistical tests were two-sided.
